# Safety evaluation of a novel topical combination of esafoxolaner, eprinomectin and praziquantel, in reproducing female cats

**DOI:** 10.1051/parasite/2021016

**Published:** 2021-04-02

**Authors:** Eric Tielemans, Heidi Erasmus, Mandie Momberg, Anthony Pfefferkorn, Norba Targa, Jaya Chilakapati, Aradhana Gupta

**Affiliations:** 1 Boehringer Ingelheim Animal Health 29 Avenue Tony Garnier 69007 Lyon France; 2 Clinvet International (Pty) Ltd. P.O. Box 11186 Universitas 9321 Bloemfontein Republic of South Africa; 3 Boehringer Ingelheim Animal Health 3239 Satellite Blvd Duluth 30096 GA USA

**Keywords:** Cat, Reproduction, Esafoxolaner, Eprinomectin, Praziquantel, Safety

## Abstract

NexGard^®^ Combo, a novel topical endectoparasiticide product for cats, is a combination of esafoxolaner, eprinomectin and praziquantel. The safety of this novel combination administered to females during reproduction and lactation was evaluated per analysis of breeding parameters and adverse reactions observed on females and offspring. Females with successful breeding history were randomized to three groups, a placebo group and groups treated with the novel formulation at 1× or 3× multiples of the maximum exposure dose. Females were dosed at 28-day intervals, at least twice before mating, then during a period including mating, pregnancy, whelping and 56 days of lactation. In the placebo, 1× and 3× groups, 10, 9 and 10 females, respectively completed the study (nine, seven and nine females achieved pregnancy), and were dosed 7.1 times on average. Breeding parameters included success of mating, success of gestation, length of gestation, abortion rate, number of live, dead and stillborn kittens at birth, number of kittens with abnormalities, weight of kittens after birth and at weaning, growth of kittens, proportion of male and female kittens, and proportion of kittens born alive and weaned. No significant adverse reactions related to the novel combination were observed on females and on kittens; no significant and adverse effects on breeding parameters were observed.

## Abstract (fr)

NexGard^®^ Combo, un nouvel endectoparasiticide topique pour chats, est une combinaison d’esafoxolaner, d’éprinomectine et de praziquantel. La sécurité de cette nouvelle association administrée aux chattes pendant la reproduction et la lactation a été évaluée par analyse des paramètres d’élevage et des effets indésirables observés sur les femelles et les descendants. Les chattes ayant des antécédents de reproduction réussie ont été randomisées en trois groupes, un groupe placebo et des groupes traités avec la nouvelle formulation à des multiples de 1× ou 3× la dose d’exposition maximale. Les femelles ont reçu des doses à 28 jours d’intervalle, au moins deux fois avant l’accouplement, puis pendant une période comprenant l’accouplement, la gestation, la mise bas et 56 jours de lactation. Dans les groupes placebo, 1× et 3×, repectivement dix, neuf et dix chattes ont terminé l’étude (neuf, sept et neuf chattes ont été gestantes) et ont été traitées 7,1 fois en moyenne. Les paramètres d’élevage comprenaient le succès de l’accouplement, le succès de la gestation, la durée de la gestation, le taux d’avortement, le nombre de chatons vivants, morts et mort-nés à la naissance, le nombre de chatons présentant des anomalies, le poids des chatons après la naissance et au sevrage, la croissance des chatons, la proportion de chatons mâles et femelles et la proportion de chatons nés vivants et sevrés. Aucun effet indésirable significatif lié à la nouvelle association n’a été observé chez les femelles et les chatons et aucun effet indésirable significatif sur les paramètres d’élevage n’a été observé.

## Introduction

NexGard^®^ Combo, a novel endectoparasiticide topical product for cats, is a combination of esafoxolaner, eprinomectin and praziquantel, aimed at the control of infestations by arthropods, and the treatment of infections by nematodes and cestodes, respectively. Esafoxolaner is a novel compound from the isoxazoline class, it is the purified and active (S)-enantiomer of afoxolaner, the racemic form. Afoxolaner has been used safely for years in oral acaricide and insecticide products for dogs (NexGard^®^ and NexGard SPECTRA^®^, Boehringer-Ingelheim). Eprinomectin and praziquantel are both well-known compounds in veterinary medicine and in cats [6, 7, 13, 14, 20, 24], with many years of safe and effective use.

The target animal safety of this novel product was evaluated in three studies designed in accordance with the requirements of the International Cooperation on Harmonisation of Technical Requirements for Registration of Veterinary Medicinal Products, VICH Guideline 43 [27], and which demonstrated safety of NexGard^®^ Combo in kittens following repeated topical administrations at 1X, 3X and 5X, and following oral ingestion at 1X the maximum exposure [12].

The potential effects of repeated administrations of NexGard^®^ Combo in reproducing female cats, on breeding parameters, as well as on their offspring were investigated in a specific safety study, described in this manuscript.

## Materials and methods

### Ethics

All animal procedures in this study were reviewed and approved by the Sponsor’s and CRO’s Institutional Animal Care and Use committees, and were in compliance with all applicable sections of the Final Rules of the Animal Welfare Act regulations (9 CFR).

### Test/control articles

The test article was NexGard^®^ Combo, a novel combination of 1.2% w/v esafoxolaner, 8.3% w/v praziquantel and 0.4% w/v eprinomectin, and contained in applicators of 0.3 mL or 0.9 mL for topical administration to cats weighing 0.8 kg – <2.5 kg (1.8–5.5 lbs) or 2.5–7.5 kg (5.6–16.5 lbs), respectively. The control article (placebo) was mineral oil.

This study was conducted with a placebo-control group, dosed with 1.125 mL/kg mineral oil, and two test article groups dosed with 1× (0.375 mL/kg) and 3× (1.125 mL/kg) the maximum exposure dose of the novel product. The delivered doses of active ingredients were 4.5, 31.1 and 1.5 mg/kg (1×) and 13.5, 93.4, and 4.5 mg/kg (3×), for esafoxolaner, praziquantel, and eprinomectin, respectively.

### Study design

The study design referred to recommendations of VICH GL 43 “Target Animal Safety – Pharmaceuticals: Target Animal Safety for Veterinary Pharmaceutical Products”, July 2008 and was conducted in accordance with the Organization for Economic Co-operation and Development (OECD) Principles of Good Laboratory Practice (Revised 1997, issued January 1998) ENV/MC/CHEM(98)17, the application of the OECD Principles of GLP to the Organisation and Management of Multi-site studies ENV/JM/MONO(2002)9 and the Directive 2004/10/EC of the European Parliament and of the Council of 11 February 2004.

This study was a negative-controlled, blinded, clinical, safety study using a completely randomized design.

Thirty-two Domestic Short-hair (DSH), purpose-bred female cats, aged 1.7–5.8 years, weighing 2.3–4.2 kg and with a successful breeding history of one (11 females) or two (21 females) normal and healthy litters were included in the study. Eight male cats that had sired at least one normal and healthy litter were used for breeding purposes, but otherwise were not dosed and not evaluated.

The 32 females were randomly allocated to the three treatment groups, resulting in 11, 10, and 11 females forming the control, 1× and 3× groups, respectively.

Animals were acclimated to study conditions for at least 7 days before the first treatment application.

Females were dosed with the test or control article at 28-day intervals during the full study: twice before co-housing with the male, until pregnancy diagnosis, during pregnancy, whelping and until weaning of kittens at the end of a 56-day lactation period (dosing was avoided during the 5 days following whelping, in which case the female was dosed 7 ± 1 days after whelping, and the original dosage schedule at 28-day intervals was resumed thereafter).

After two monthly doses, the female was co-housed with an appropriate male, based on compatibility and an inbreeding coefficient of less than 25% with the female. Up to three females were co-housed with a male, until confirmed pregnant. Pregnancy diagnosis was made by ultrasound scan, 21 days after start of co-housing with the male and repeated weekly until confirmed pregnant. Once confirmed pregnant, the female was moved in the maternity yard and single housed, and 2–3 extra scans were performed while the gestation progressed, to monitor pregnancy. Females that did not achieve pregnancy after 90 days of co-housing with the male were moved to single housing and scanned weekly for another 3 weeks for final pregnancy status diagnosis.

Females, males and kittens were observed for health at least twice daily until the end of the study. Females were also observed hourly for 6 h after each test/control article application. A physical examination was performed for all females and males before inclusion, and for females, at the end of the lactation period. A physical examination was performed for kittens weekly, from 7 days of age to weaning at 56 days.

Females were weighed every 4 weeks, within 5 days of each treatment application, 7 days after whelping and at the end of the lactation period. Kittens were weighed once a week 7 times, from the age of 7–56 days. At the end of the study (i.e. end of lactation/weaning for females and kittens), animals were returned to the facility colony.

Any animal that was born dead, died at birth, after birth or was euthanized was necropsied. Representative samples of tissues were collected for all animals and processed for histopathologic examination. Kittens less than 3 weeks old had their whole body bisected and gross lesions (when applicable) tagged. Kittens 3 weeks and older and females had the following tissues collected: gross lesions (when applicable), whole brain, cervical spine with spinal cord (including C1–C2), sciatic nerve, stomach, duodenum, jejunum, ileum, cecum, colon, kidneys, liver, heart, lungs, and for adult females only, mammary gland, ovaries, uterus and vagina. All specimens were examined microscopically by an ACVP Board-certified pathologist and were peer-reviewed by a second ACVP Board-certified pathologist.

### Data analysis

In addition to health observations of females and kittens, and anatomic pathology evaluations of females and kittens that died or were euthanized, breeding parameters were analyzed to assess any interference of the novel formulation. Breeding parameters included success of mating and gestation, length of gestation, abortion rate, number of live/dead kittens at birth, number of kittens born with abnormalities, weight of kittens 1 week after birth and at weaning and growth during lactation, proportion of male and female kittens, and proportion of kittens born alive and weaned. Breeding parameters, statistical analyses, and results are described in Table 1.

**Table 1:** Breeding parameters, statistical analyses, and results.

	Group 1	Group 2	Group 3
Mean gestation length^a^: days (SD)	45.7 (3.35)	45.3 (2.21)	45.8 (2.05)
*p*-value: one-way ANOVA with a treatment effect		0.7771	0.9296
No. of mating pairs	10	9	10
No. of pairs confirmed to have mated	9	7	9
Mating index	0.90	0.78	0.90
*p*-value vs. Group 1. Fisher’s exact test		0.5820	1.0000
No. of mating pairs	10	9	10
No. of confirmed pregnancies	9	7	9
Conception index	0.90	0.78	0.90
*p*-value vs. Group 1. Fisher’s exact test		0.5820	1.0000
No. of confirmed pregnancies	9	7	9
No. of females with live kittens	9	7	9
Gestational index	1.00	1.00	1.00
*p*-value vs. Group 1. Fisher’s exact test		0.5820	1.0000
No. of confirmed pregnancies	9	7	9
No. of females who had an abortion	0	0	0
Abortion rate	0.00	0.00	0.00
*p*-value vs. Group 1. Fisher’s exact test	Data structure unsupported by Fischer’s exact test		
No. of litters	9	7	9
No. of kittens born^b^ (Min, Max) – Average per litter	34 (2, 6) – 3.8	38 (3, 8) – 5.4	53 (3, 8) – 5.9
*p*-value: one-way ANOVA with a treatment effect		0.0486	0.0092
No. of kittens born alive^c^ (Min, Max)	33 (2, 6)	36 (3, 8)	51 (3, 8)
*p*-value: one-way ANOVA with a treatment effect		0.0821	0.0150
No. of stillborn kittens^d^	1	2	2
Proportion	0.03	0.03	0.04
*p*-value vs. Group 1. Fisher’s exact test		1.0000	1.0000
No. of kittens born with an abnormality^e^	1	2	3
Proportion	0.03	0.03	0.06
*p*-value vs. Group 1. Fisher’s exact test		1.0000	1.0000
No. of litters with at least one abnormality	1	2	2
Proportion	0.11	0.29	0.22
*p*-value vs. Group 1. Fisher’s exact test		1.0000	1.0000
No. of kittens weaned (56 days after birth)	27	31	50
Weaning index (vs. n° of kittens born alive)	0.82	0.86	0.98
*p*-value vs. Group 1. Fisher’s exact test		0.7465	0.0134
No. of female kittens born (proportion)	18 (0.53)	19 (0.50)	29 (0.55)
No. of male kittens born (proportion)	16 (0.47)	15 (0.39)	23 (0.43)
Sexing omitted (proportion)	0 (0.0)	4 (0.11)	1 (0.02)
*p*-value vs. Group 1. Fisher’s exact test		0.1798	0.8950
Mean bodyweight of kittens on Day 7: kg (SD)	0.208 (0.032)	0.179 (0.027)	0.180 (0.025)
*p*-value: one-way ANOVA with a treatment effect		<0.0001	<0.0001
Mean bodyweight of kittens on Day 56: kg (SD)	0.807 (0.154)	0.686 (0.102)	0.648 (0.104)
*p*-value: one-way ANOVA with a treatment effect		0.0002	<0.0001
Mean growth of kittens from Day 7 to Day 56: kg	0.599	0.506	0.477
*p*-value: one-way ANOVA with a treatment effect		0.0030	<0.0001

Group 1: Placebo (mineral oil) at 1.125 mL/kg; Group 2: NexGard^®^ Combo at 0.375 mL/kg; Group 3: NexGard^®^ Combo at 1.125 mL/kg.

a Gestation length calculated from the day of pregnancy diagnosis to the day of whelping.

b Includes kittens born alive, found dead on whelping day and stillborn.

c Includes kittens born alive, found dead on whelping day, excludes stillborn.

d Stillborn = dead on whelping day, without a single pulmonary inspiratory flow (as determined by lung histology).

e Includes stillborn.

## Results

Three females were removed from the study after mating but before whelping and were not replaced: one female from the 1× group was inadvertently co-housed with a genetically inappropriate male leading to an inbreeding coefficient of more than 25% after having received three treatments; one pregnant female from the control group that had received four treatments was humanely euthanized and necropsied (together with one fetus that was collected) after health deterioration, and was diagnosed infected with Feline Infectious Peritonitis (FIP) (histology did not reveal any abnormality for the fetus); one not-pregnant female from the 3× group was diagnosed with pyometra after having received three treatments. None of these adverse events were considered related to the application of the test or control article. Consequently, 10, 9 and 10 females in the control, 1× and 3× groups, respectively, completed the study and were dosed on average 6.3 (6–7), 6.7 (6–8) and 7.3 (6–9) times.

Amongst the 29 females that completed the study, four females (one, two and one females from the control, 1× and 3× groups, respectively), did not become pregnant after 90 days of co-housing with a male. All 25 females diagnosed pregnant (nine, seven and nine females from the control, 1× and 3× groups, respectively), remained so until whelping. All whelpings were uneventful and no manual, chemical or surgical assistance was required.

Amongst 125 kittens born, 108 kittens remained alive and in good health until weaning. Seventeen kittens (7, 7 and 3 from the control, 1X and 3X groups, respectively) did not survive until weaning: six kittens (two from each group) were found dead on the day of whelping, of which four were determined stillborn by histology, i.e. without a single pulmonary inspiratory flow; three kittens (2 and 1 from the control and 1X groups, respectively) were euthanized on whelping day because of circulatory damages caused by abnormal positioning of the umbilical cord during the whelping process; six kittens (3 and 3 from the control and 1× groups, respectively) were found dead between 1 and 3 days of age; one kitten from the 3× group was born with an incomplete closure of the cranial fontanelle causing hydrocephalus which prompted euthanasia at 25 days of age; one kitten from the 1× group was euthanized at 46 days of age for locomotor paralysis of traumatic origin. Full necropsy and histopathological examination were performed for all 17 dead and euthanized kittens. None of the macroscopic or microscopic examinations revealed any abnormalities that were considered test or control article-related.

Other minor health abnormalities such as diarrhea, constipation, eye/nasal discharge and alopecia affected some females, males and kittens and were not considered test or control article-related. Concurrent medications were used for some of these conditions and included topical or systemic antibiotics, non-steroidal anti-inflammatories, vitamins or prescription feed, and were not considered to have any impact on the study. The mean group bodyweights of females before first treatment, 7 days after whelping and on Day 56 at the end of the lactation period were not significantly different.

Breeding parameters are detailed in Table 1.

The verified breeding parameters were consistent with those described in the scientific literature for the feline species [9, 18, 22].

No significant differences were seen between the control and the NexGard^®^ Combo treated groups with regards to gestation length, mating/conception index (pregnant females per mating pairs), gestational index (females with live kittens per pregnant females), abortion rate, number of still born kittens, number of kittens with abnormalities, and sex ratio of kittens.

The number of kittens born per litter were significantly higher in the NexGard^®^ Combo treated groups than in the control group, and the number of kittens born alive per litter were significantly higher in the NexGard^®^ Combo 3× group than in the control group. The proportion of kittens born alive and alive at weaning at 56 days of age (weaning index) was significantly higher in the NexGard^®^ Combo 3× group than in the control group. The average bodyweight of kittens at 7 days of age and at 56 days of age, as well as their growth between these 2 days were significantly lower in the NexGard^®^ Combo treated groups than in the control group.

## Discussion

There is no explanation as to why the females treated with NexGard^®^ Combo, and especially at the 3× dosage produced a higher number of kittens and live kittens at birth and at weaning, than the placebo treated females. No comparable data exist about any of the three active ingredients taken separately. Fluctuations in litter size are expected in felines, larger litter sizes at the 1× and 3× dosages are not considered treatment-related or adverse. The lower bodyweights and growth of kittens in the NexGard^®^ Combo treated groups compared to the control group can be explained by the significant larger litter sizes in the treated groups, which directly affected the bodyweight of newborn kittens [18] and growth of kittens [9], as larger litters place a burden on themselves, namely with regards to competition for nutrients. The bodyweights and growth of kittens in the NexGard^®^ Combo treated groups were nevertheless consistent with bodyweights described in the literature [9].

In the context of this study, NexGard^®^ Combo administered monthly to breeding female cats at up to 3 times the maximum exposure dose, during reproduction and lactation, did not have any impact on breeding parameters and did not cause significant adverse reactions to females and kittens.

Parasitism is an important medical condition of the feline species, which is permanently exposed to ecto- and endo-parasites worldwide, at variable levels depending on local epidemiology, and environmental and living conditions. Epizootiological studies performed in several regions of the world on healthy domestic or shelter/stray cats have demonstrated significant prevalence of parasites in the feline species; with endoparasite infections consistently ranging from approximately 20–50% [3, 17, 19, 26]; flea infestations from 15% to 100% [3, 16, 23]; and ear mite infestation from 17% to 26% [3, 25]. It is therefore important to provide permanent parasiticide therapeutic solutions to feline veterinary medicine, with the possibility to treating females during their 4 months’ period of reproduction and lactation, to protect them from their local parasitic risks, but also to minimize exposure to kittens, which are highly sensitive to some parasites because of their immature immune system or their lack of protective behavior.

For example, *Toxocara cati*, the most prevalent gastro-intestinal nematode in cats [2, 3, 5, 11, 19, 21, 26], has a significant prevalence in young kittens [1, 10], in which the lactogenic route is an important path of infection, alongside direct infection by ingestion of environmental infective eggs [4, 8]. It is also important to continuously control the environmental flea burden by keeping females on regular treatment, as kittens easily become infested by newly emerged fleas and have little or poorly efficient grooming behavior to minimize their ectoparasitic load. Similar principles can be applied to *Otodectes cynotis* ear mites, which are highly prevalent in the feline population and regularly infest young kittens [15, 25], with the female being a possible source of infestation as the acarian uses direct contact as a route of infestation.

## Competing interest

The work reported herein was funded by Boehringer-Ingelheim. Some of the authors are current employees of Boehringer-Ingelheim Animal Health. Other than that, the authors declare no conflict of interest. This document is provided for scientific purposes only. Any reference to a brand or trademark herein is for information purposes only and is not intended for any commercial purposes or to dilute the rights of the respective owners of the brand(s) or trademark(s). NexGard^®^ is a registered trademark of the Boehringer Ingelheim Group.
